# Dementia prevention in memory clinics: recommendations from the European task force for brain health services

**DOI:** 10.1016/j.lanepe.2022.100576

**Published:** 2023-01-31

**Authors:** Giovanni B. Frisoni, Daniele Altomare, Federica Ribaldi, Nicolas Villain, Carol Brayne, Naaheed Mukadam, Marc Abramowicz, Frederik Barkhof, Marcelo Berthier, Melanie Bieler-Aeschlimann, Kaj Blennow, Andrea Brioschi Guevara, Emmanuel Carrera, Gaël Chételat, Chantal Csajka, Jean-François Demonet, Alessandra Dodich, Valentina Garibotto, Jean Georges, Samia Hurst, Frank Jessen, Miia Kivipelto, David J. Llewellyn, Laura McWhirter, Richard Milne, Carolina Minguillón, Carlo Miniussi, José Luis Molinuevo, Peter M. Nilsson, Alastair Noyce, Janice M. Ranson, Oriol Grau-Rivera, Jonathan M. Schott, Alina Solomon, Ruth Stephen, Wiesje van der Flier, Cornelia van Duijn, Bruno Vellas, Leonie N.C. Visser, Jeffrey L. Cummings, Philip Scheltens, Craig Ritchie, Bruno Dubois

**Affiliations:** a Memory Center, Department of Rehabilitation and Geriatrics, University Hospitals and University of Geneva Geneva, Switzerland; b Institut de la Mémoire et de la Maladie d’Alzheimer, IM2A, Groupe Hospitalier Pitié-Salpêtrière, Sorbonne Université, Paris, France; c Institut du Cerveau et de la Moelle Épinière, UMR-S975, INSERM, Paris, France; d Cambridge Public Health, University of Cambridge, Cambridge, UK; e Division of Psychiatry, University College London, London, UK; f Genetic Medicine, Diagnostics Dept, University Hospitals and University of Geneva, Geneva, Switzerland; g Radiology & Nuclear Medicine, Amsterdam University Medical Centers, Amsterdam, the Netherlands; h Queen Square Institute of Neurology, University College London, London, UK; i Unit of Cognitive Neurology and Aphasia, Centro de Investigaciones Médico-Sanitarias (CIMES), University of Malaga, Malaga, Spain; j Leenaards Memory Centre, Department of Clinical Neurosciences, University Hospital of Lausanne (CHUV), Lausanne, Switzerland; k Infections Disease Service, University Hospital of Lausanne (CHUV), Lausanne, Switzerland; l Clinical Neurochemistry Laboratory, Institute of Neuroscience and Physiology, University of Gothenburg, Sahlgrenska University Hospital, Mölndal, Sweden; m Faculty of Psychology and Educational Sciences, University of Geneva, Geneva, Switzerland; n Stroke Center, Department of Clinical Neurosciences, University Hospitals and University of Geneva, Geneva, Switzerland; o Normandie University, UNICAEN, INSERM, U1237, PhIND Physiopathology and Imaging of Neurological Disorders, Cyceron, Caen, France; p Center of Research and Innovation in Clinical Pharmaceutical Sciences, University Hospital and University of Lausanne, Lausanne, Switzerland; q French Clinical Research Infrastructure Network, INSERM, University Hospital of Toulouse, France; r Center for Mind/Brain Sciences (CIMeC), University of Trento, Rovereto, Italy; s Division of Nuclear Medicine and Molecular Imaging, University Hospitals of Geneva and NIMTLab, University of Geneva, Geneva, Switzerland; t Alzheimer Europe, Luxembourg; u Institute for Ethics, History, and the Humanities, Faculty of Medicine, University of Geneva, Geneva, Switzerland; v Department of Psychiatry, Faculty of Medicine and University Hospital Cologne, University of Cologne, Cologne, Germany; w German Center for Neurodegenerative Diseases (DZNE), Bonn-Cologne, Germany; x Excellence Cluster Cellular Stress Responses in Aging-Related Diseases (CECAD), Medical Faculty, University of Cologne, Germany; y Division of Clinical Geriatrics, Center for Alzheimer Research, Department of Neurobiology, Care Sciences and Society, Karolinska Institutet, Stockholm, Sweden; z Institute of Public Health and Clinical Nutrition, University of Eastern Finland, Kuopio, Finland; aa Theme Aging, Karolinska University Hospital, Stockholm, Sweden; ab The Ageing Epidemiology Research Unit, School of Public Health, Imperial College London, London, UK; ac College of Medicine and Health, University of Exeter, UK; ad Alan Turing Institute, Exeter, UK; ae Centre for Clinical Brain Sciences, University of Edinburgh, Edinburgh, Scotland, UK; af Engagement and Society, Wellcome Connecting Science, Hinxton, UK; ag Barcelonaβeta Brain Research Center (BBRC), Pasqual Maragall Foundation, Barcelona, Spain; ah IMIM (Hospital del Mar Medical Research Institute), Barcelona, Spain; ai CIBER Fragilidad y Envejecimiento Saludable (CIBERFES), Madrid, Spain; aj Centre for Medical Sciences (CISMed), University of Trento, Rovereto, Italy; ak H. Lundbeck A/S, Denmark; al Department of Clinical Science, Lund University, Sweden; am Department of Internal Medicine, Skåne University Hospital, Malmö, Sweden; an Preventive Neurology Unit, Wolfson Institute of Population Health, Queen Mary University of London, London, UK; ao Dementia Research Centre, UCL Queen Square Institute of Neurology, London, UK; ap Institute of Clinical Medicine, University of Eastern Finland, Kuopio, Finland; aq Division of Clinical Geriatrics, NVS, Karolinska Institutet, Stockholm, Sweden; ar Alzheimer Center Amsterdam, Department of Neurology, Amsterdam Neuroscience, Amsterdam UMC, Amsterdam, the Netherlands; as Amsterdam Neuroscience, Neurodegeneration, Amsterdam, the Netherlands; at Epidemiology and Data Science, Vrije Universiteit Amsterdam, Amsterdam UMC location VUmc, Amsterdam, the Netherlands; au Department of Epidemiology, Erasmus University Medical Center, Rotterdam, the Netherlands; av Clinical Trial Service Unit and Epidemiological Studies Unit, Nuffield Department of Population Health, University of Oxford, Oxford, UK; aw Gerontopole and Alzheimer's Disease Research and Clinical Center, Toulouse University Hospital, Toulouse, France; ax Department of Medical Psychology, Amsterdam Public Health Research Institute, Amsterdam UMC, Amsterdam, the Netherlands; ay Chambers-Grundy Center for Transformative Neuroscience, Department of Brain Health, School of Integrated Health Sciences, University of Nevada, Las Vegas, NV, USA; az EQT Life Sciences, Amsterdam, the Netherlands; ba Centre for Clinical Brain Sciences, University of Edinburgh, Edinburgh, UK

**Keywords:** Dementia, Prevention, Memory clinic, Risk assessment, Risk communication, Risk reduction, Cognitive enhancement

## Abstract

Observational population studies indicate that prevention of dementia and cognitive decline is being accomplished, possibly as an unintended result of better vascular prevention and healthier lifestyles. Population aging in the coming decades requires deliberate efforts to further decrease its prevalence and societal burden. Increasing evidence supports the efficacy of preventive interventions on persons with intact cognition and high dementia risk. We report recommendations for the deployment of second-generation memory clinics (Brain Health Services) whose mission is evidence-based and ethical dementia prevention in at-risk individuals. The cornerstone interventions consist of (i) assessment of genetic and potentially modifiable risk factors including brain pathology, and risk stratification, (ii) risk communication with ad-hoc protocols, (iii) risk reduction with multi-domain interventions, and (iv) cognitive enhancement with cognitive and physical training. A roadmap is proposed for concept validation and ensuing clinical deployment.

## Introduction

Despite the global increase of the prevalence of dementia, the age-specific incidence of dementia is decreasing,^1^ indicating that the prevention of dementia is not only possible, but already under way. While this outcome has so far been an indirect result of societal changes and provides emphasis for societies to address inequalities and life-course influences on brain health, there is a demand to develop individualized services and evidence that targeted interventions might further decrease dementia risk.^2^ This is the focus of this clinically oriented paper, given that many clinicians are finding their advice and expertise is sought on an individual basis for proactive, prospective risk reduction programs. These have been advocated, among others, by the 2019 WHO guidelines on risk reduction of cognitive decline and dementia^3^ and by initiatives driven by the European Academy of Neurology,^4^ the Scottish government and Alzheimer Scotland,^5^ Karolinska Institute (Kivipelto, personal communication), and the German, Norwegian, and Polish governments.^6^

The global prevalence of persons at risk for cognitive impairment or dementia due to AD pathology has been estimated at 315 millions.^7^ Although a number of clinical trials on modifiable risk factors have failed to achieve their primary endpoints,^8^ a few recent trials in dementia-free participants have suggested that cognitive performance can be efficiently boosted with multi-domain interventions in at-risk persons,^9^^,^^10^ indicating that the risk of dementia and cognitive impairment might be reduced with multiple interventions on life-styles and vascular risk in specific patient groups.^2^^,^^11^, ^12^, ^13^ Preliminary observations also point to a potential beneficial effect of non-invasive brain stimulation (NIBS) on cognitive outcomes.^14^^,^^15^

Diagnosis and management for patients with cognitive complaints and concerns in high income countries is currently delivered by memory clinics. The clinical and organizational features are illustrated in Table 1. The health offer in memory clinics consists of the clinical and instrumental evaluation, diagnosis, staging, treatment, and rehabilitation. In line with the growing ability to seek an etiological diagnosis,^16^ many memory clinics employ biomarkers and genetic testing to achieve a taxonomical classification as close as possible to neuropathology. The challenges of diagnostic communication in memory clinics include those typical of progressive and disabling diseases. Diagnosis is followed by prognosis and non-pharmacologic and pharmacologic treatment aimed to temporarily delay disability and relieve psychological distress. Most procedures and interventions are reimbursed by health care payers.

**Table 1:** Synopsis of analogies and differences between traditional memory clinics and brain health services.

	Memory clinics	Brain health services
**Context**	Outpatient facilities in the context of neurology, geriatric, or psychiatric services	Clinical spinoffs of currently active memory clinics, specific health care offering within current memory clinics, or independent new services
**Target population**	Cognitively impaired individuals (mild cognitive impairment, dementia) possibly with behavioural and psychological symptoms of dementia	Cognitively unimpaired individuals, potentially at risk (subjective cognitive decline, family history, “worried well”)
**Health offer**	Evaluation, disease diagnosis, staging, prognosis, treatment, rehabilitation, and psychological support	Risk profiling, risk communication, risk reduction, and cognitive enhancement
**Workup**	• Basic dementia workup (history, cognitive screening, neurological exam, brain MRI, optional electroencephalography) • Neuropsychological testing • Aβ42, tau and p-tau in cerebrospinal fluid • Positron emission tomography with 18 F-fluorodeoxyglucose (FDG), amyloid or tau tracers, dopamine imaging	
	• Diagnostic genetic testing (APP, PS1, PS2, C9ORF72, etc.)	• Potentially modifiable risk factor assessment • Genetic risk factor assessment (*APOE* and common low-risk genes)
**Personnel**	• Dementia specialists (neurologists, geriatricians, psychiatrists) • Psychologists (neuropsychologists, psychotherapists, speech therapists) • Physical and occupational therapists	
**Communication**	• Etiologic diagnosis (e.g. Alzheimer's disease, dementia with Lewy bodies, limbic predominant age-associated TDP-43 encephalopathy, etc.) • Prognosis • Efficacy and adverse effects of treatments	• Concept of risk • Potential of prevention
**Interventions**	• Driven by etiologic diagnosis and staging • Aimed at reducing and delaying disability • Drugs: symptomatic (cholinesterase inhibitors, memantine, monoclonal antibodies where available) • Randomized drug trials: symptomatic and disease modifying drugs aimed at delaying disability and reversing pathology	• Driven by individual cumulative risk • Multi-domain interventions based on lifestyles (cognitive and physical interventions) and targeting vascular risk • Randomized drug trials: drugs targeting risk factors aimed at reducing the incidence of cognitive impairment and dementia • Possibly non-invasive brain stimulation • Possibly drugs active on amyloid, tau, neuroinflammation, oxidative stress, brain metabolism, etc.
**Technological platform**	• Neuropsychological test batteries • Imaging (Magnetic resonance imaging and Positron emission tomography scanners) • Scales for functional and behavioral assessment • Next generation sequencing for autosomal dominant mutations	
	• Fully automated platform for CSF biomarkers (e.g. Elecsys or Lumipulse) • Electroencephalography	• Fully automated (e.g. Elecsys) or semi-automated ultra-sensitive (e.g. SIMOA Single molecule array) platforms for blood biomarkers • Real-time polymerase chain reaction for *APOE* genotyping and polygenic risk scores • Digital and telehealth services
**Reimbursement**	• Health care payers	• Research funds, out-of-pocket money, integrative insurance • Health care payers may reimburse with accumulation of evidence on dementia risk reduction

The BHS items have different levels of clinical readiness, as detailed in the text.

## Current challenges in clinical practice

Current memory clinics are not designed for and often not well placed to evaluate and treat unimpaired individuals with cognitive complaints or concerns who may or may not develop disease in the future. These are attending with increasing frequency and have been designated as having “subjective cognitive decline” or being “worried well” (for definitions see panel 1).^17^ While memory clinics have little to offer them beyond reassurance about their current cognitive status and recommendations on healthy lifestyles, what this population is asking for is an estimate of their dementia risk beyond the well-known healthy lifestyles, support to reduce their risk of developing cognitive impairment and dementia, and sometimes cognitive augmentation. In analogy to the vascular risk factor management model,^18^ the following actions are required: detection of all known risk factors and categorization of persons into risk strata (risk assessment), communication of the risk, and engagement in risk reduction or cognitive enhancement interventions. None of this is part of the toolkit of standard memory clinics.

**Panel 1**

**Search strategy and selection criteria**

References for this Health Policy paper were identified by searches of PubMed between 2010 and March 2022, and references from relevant articles. Search criteria are described in the original papers this paper is based on (references #4 to #9). The search terms were:

- For measuring dementia risk and risk profiling: "dementia risk" and "risk of dementia"
- For risk communication: "communication of risk" and "risk communication"
- For personalized prevention: “risk reduction”, “multidomain interventions”, and “prevention trials”
- For cognitive enhancement: “cognitive training”, “meditation”, “physical training”, “non-invasive brain stimulation”, “transcranial stimulation”, “cognitive enhancers”, “nootropics”, and “nutritional supplements”.

Restriction of the search to the title field was used as a strategy to narrow it down to the most pertinent articles. Reviews were used as means to identify original research articles. Only studies on persons with no cognitive impairment or subjective cognitive decline were selected. There were no language restrictions. The final reference list was generated based on relevance to the topics covered in this Review.

**Definitions**

- **Alzheimer’s disease:** cognitive impairment with evidence of amyloid and tau deposition in the brain according to the International Working Group (IWG) criteria of Dubois et al., 2021.^25^ The diagnosis of Alzheimer's disease should be made only in persons with cognitive impairment. Cognitively unimpaired persons with in-vivo biomarker evidence of Alzheimer's disease pathology are considered “at risk” of progression to cognitive impairment and Alzheimer's dementia. This diagnostic framework has been developed for clinical and research use, includes the notion of risks associated with vascular disorders, and is in line with the societal narrative of Alzheimer's disease.^25^
- **Alzheimer's pathology:** hallmarks of the disease detected in the brains of patients at autopsy (β-amyloid deposits and neurofibrillary tangles). Amyloid PET and tau PET are accurate in-vivo proxies for moderate to frequent plaques and advanced tau deposition (Braak stage V-VI). Measurements of amyloid beta-42 and phosphorylated tau in the cerebrospinal fluid are early and accurate markers of brain amyloid and tau deposition.
- **Biomarker:** an objectively measurable substance, characteristic, or other parameter of a biological process that enables assessment of disease risk or prognosis and provides guidance for diagnosis or monitoring of treatment.
- **Cognitive impairment:** a statistical construct denoting performance on cognitive tests consistently below age- and education-specific norms.
- **Cognitive (repeated practice and strategic learning) and physical training:** behavioral interventions aiming to protect brain function against age-related decline. Repeated practice consists of the frequent rehearsal of a set of actions aimed to restore a cognitive function (e.g., play a video game or practice mindfulness to train attention). Strategic learning consists in optimizing daily living functioning to compensate for an impaired cognitive function (e.g., using mnemonics and/or external aids for memory loss). Physical training consists in practicing sustained physical activity with a structured exercise program (e.g., warming up, aerobic exercise, cool down with stretching/relaxation).
- **Dementia:** syndrome with acquired progressive cognitive impairment severe enough to affect daily activities. Generally, a more severe stage of Mild cognitive impairment in persons with progressive cognitive deterioration.
- **Functional cognitive disorders:** a range of overlapping psychiatric conditions in which cognitive complaints and concerns can present in isolation or be part of anxiety or depression, dissociative seizures and functional movement disorders, chronic fatigue syndrome, fibromyalgia, and dissociative cognitive states (e.g. dissociative amnesia, fugue, Ganser syndrome).^51^
- **Mild cognitive impairment:** a clinical construct consisting of acquired cognitive impairment without functional limitation with heterogeneous presentations and underlying pathologies (Alzheimer's, hippocampal sclerosis, frontotemporal degeneration, or Lewy body disease) or sometimes normal age-related changes.^69^
- **Neurodegeneration:** progressive loss of structure and function of neurons, including loss of synapses and death of neurons. Positron emission tomography with 18 F-fluorodeoxyglucose (FDG) and volumetric magnetic resonance imaging are markers of neurodegeneration in a number of neurodegenerative dementias, and dopamine imaging with [^123^I]FP-CIT single photon emission tomography is a marker of neurodegeneration of the striato-nigral pathway in dementias with parkinsonism.
- **Non-invasive brain stimulation (NIBS):** techniques aimed to enhance or inhibit synaptic transmission and functional connectivity. They consist of repetitive transcranial magnetic stimulation (rTMS) and low intensity transcranial direct or alternate current stimulation (tDCS, tACS).
- **Risk assessment:** a systematic approach to collecting information from individuals that identifies risk factors, provides individualized feedback, and links the person with at least one intervention to promote health, sustain function and/or prevent disease.^70^ Risk assessment should be comprehensive, quantitative, and hierarchical.
- **Subjective cognitive decline:** self-perceived decline in any cognitive domain over time with normal scores on cognitive tests.^17^
- **Worried well:** individuals who do not report subjective cognitive decline and achieve normal scores on cognitive tests but who are concerned about their brain health because of a positive family history of dementia, professional reasons (physicians, nursing home personnel) or other life events.^17^
- **Worried well by proxy:** individuals who seek medical help following concerns by their family although they themselves do not report subjective cognitive decline, achieve normal scores on cognitive tests, and are not concerned about their brain health.

This paper is a guide aimed at clinicians and service providers outlining the mission, instruments, and activities of a new type of service provision through what we called Brain Health Services (BHS).^19^ It should be highlighted that the Scottish government has recently funded demonstrator sites for Brain Health Scotland's clinical services that will test the concepts outlined here^5^ and a similar model has been used in the past for currently active clinical services (e.g. National Center for Alzheimer's disease in Italy).^20^

## The memory clinic of the future

**Fig. 1:**
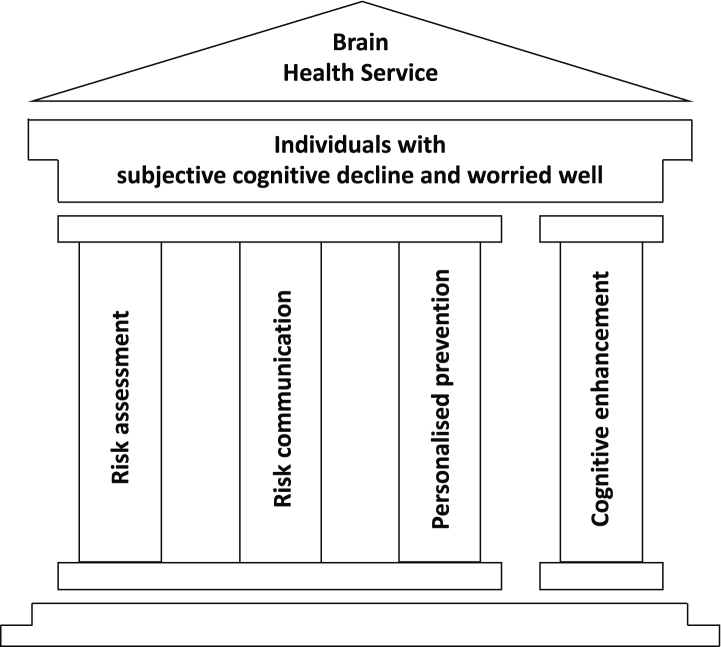
Patient population and cornerstone interventions of Brain Health Services. Risk assessment, risk communication, and personalised prevention of cognitive decline are grouped together as they take place sequentially. Cognitive enhancement is conceptually independent from interventions on risk.

**Fig. 2:**
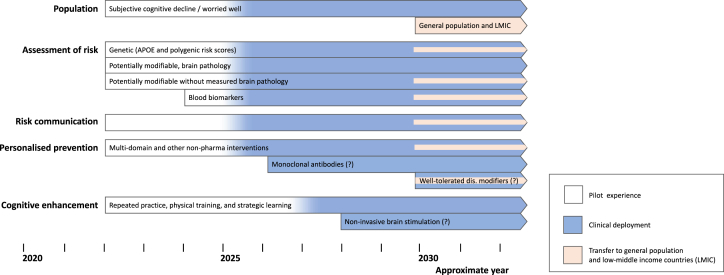
Roadmap of the hypothetical deployment of BHSs from pilot experiences to clinical deployment in the context of key scientific and technological developments. At the present time, BHSs are proposed as pilot experiences (white background) targeting persons with cognitive complaints (subjective cognitive decline) and worried-well persons in clinical outpatient settings. The four pillars of risk assessment, risk communication, personalized prevention, and cognitive enhancement comprise individual components (expanded in the text) that will be implemented over time according to their maturity. Blood biomarkers may be the first scientific and technological advancements to be implemented by BHSs. Anti-amyloid or anti-tau monoclonal antibodies and other better tolerated drugs may follow depending on the outcome of currently ongoing preventive clinical trials (see text). The transition of BHSs from pilot experiences (white color) to clinical deployment (blue color) will take place after performance evaluation based on pre-defined endpoints. We anticipate that non-invasive brain stimulation may be adopted for cognitive enhancement, if ever, at a later time. If and when the two key enablers of blood biomarkers and well tolerated drugs for secondary prevention reach maturity and after a testing period in BHSs, some components may be transferred to at-risk persons from the general population with specific attention to low-middle income countries (beige color). At that point in time, individual- and population-based interventions will co-exist. The question mark denotes uncertain successful development. Timelines in the x-axis are hypothetical and heavily dependent on scientific and technological developments.

The next sections illustrate the protocols, tools, and procedures that we propose for adoption in BHSs, whose operational pillars have been previously described^19^ and are illustrated in Fig. 1. The following sections represent a harmonized summary and manageable review of previous contributions. They also include original material and namely: a synopsis of analogies and differences between traditional memory clinics and brain health services (Table 1), an infographic of the cornerstone interventions of BHSs (Fig. 1), a synopsis of all risk factors and assessment tools (Table 2), a systematic GRADE review on training interventions for cognitive enhancement in persons with subjective cognitive decline (Supplementary Table), and a roadmap for the validation and deployment of BHSs (Fig. 2). Moreover, the table on risk communication published in Visser et al. (2021)^24^ has been deeply revised (Table 3).

**Table 2:** Dementia risk factors and dementia risk scales (adapted from Ranson et al., 2021^21^ and Frisoni et al., 2019^19^).

Risk factor	Relative risk^a^	Assessment method	Dementia risk scales^b^		
			CAIDES1	ANU-ADRIS2,S3	BDSIS4
**Genetic**					
*APOE-*ε4 heterozygous	1.9	Real-time protein chain reaction	**●**		
*APOE-*ε4 homozygous	5.3				
**Potentially modifiable without measured brain pathology**					
Early life (age <45 years)					
Less education (primary school only)	1.6	I—International Standard Classification of EducationS5	**●**	**●**	**●**
		P—Years of educationS5			
Midlife (age 45–65 years)					
Hearing loss	1.9	I—Pure tone audiometryS6 P—Whispered Voice Test,S7 speech-in-noise paradigms or self-report			
Traumatic brain injury	1.8	I—Ohio State University traumatic brain injury identification methodS8 P—Medical history, informant- or self-report		**●**	
Hypertension (>135–140/85–90)S9^,^^c^	1.6	I—Ambulatory devices, physician measurement P—Domestic devices, patient self-measurement	**●**		
Alcohol consumption (>21 units per week)	1.2	I—Quantity-frequency measures with beverage-specific assessment of time frames and binge-drinking episodesS10 P—Self report		**●**	
Obesity (body-mass index ≥30)	1.6	I—Waist circumferenceS11 and measurement of height and weight P—Body mass index based on self-report	**●**		**●**
Late life (age >65 years)					
Smoking	1.6	Self-report of smoking status (pack years, i.e. number of daily packs multiplied by number of years smoking; or current smoking status, i.e. current versus former/never smoker)		**●**	
Depression	1.9	I—Rating scales e.g. Patient Health Questionnaire (PHQ)S12 or the Hospital Depression and Anxiety ScaleS13 P—Self-report of feeling depressed or having history of diagnosed depression		**●**	**●**
Social isolation	1.6	I—Rating scales, e.g. the Lubben Social Network ScaleS14 or the Duke Social Support IndexS15 P—Self-report of social isolation		**●**	
Physical inactivity	1.4	I – Accelerometers,S16 heart rate counters,S16 smart phone,S16 or smart watch appsS16 P – Self-reported measures/questionnaires	**●**	**●**	
Diabetes	1.5	I—Fasting plasma glucose levels (≥7.0mmol/l) or HbA1c (≥6.5%), or oral glucose tolerance test to diagnose impaired glucose toleranceS17 P—Medical history, informant or self-report		**●**	**●**
Air pollution	1.1	Further research is needed to establish a practical and clinically relevant measureS18			
**Potentially modifiable, brain pathology**					
Amyloidosis	5.0 at 65 y 2.4 at 85 y^d^	I—Automated or semi-automated assay of Aβ in the CSF or amyloid PET using visual reading or centiloid quantificationS19-S23 P—Ultra sensitive assay of Aβ in plasmaS19			
Amyloidosis and tauopathy	2.8–9.1	I—As above plus p-tau in the CSF or tau PETS19,S20,S24,S25 P – As above plus p-tau in plasmaS19			
Neurodegeneration	1.6–3.1	I – Ultra sensitive assay of NfL in the CSF or plasmaS26 P—Qualitative or quantitative assessment of ventricular dilatation and medial temporal atrophy on MRIS27			
Amyloidosis and neurodegeneration	21.4 at 65 y 4.9 at 85 y^d^	See above			
Subcortical cerebrovascular disease	1.7–3.0	I—Volumetry of white matter changes and standardized scales for microbleeds on MRIS28 P—Visual rating of white matter changes, lacunes, and microbleeds on MRIS29			

Potentially modifiable risk factors without measured brain pathology are those 12 identified in 2020 by The Lancet Commission.^22^ Assessment methods are summarized, categorized into ideal (I) and practical (P), and referenced. The assessment method may differ when the risk factor is measured in the context of dementia risk scales; in this case, please refer to the original scale reference. References cited in the table can be found in the Supplementary material.

a The relative risks of potentially modifiable risk factors without measured pathology are taken from (Livingston et al., 2020^22^); these were computed taking into account communality (the variance in observed variables accounted for by common factors). The relative risks of *APOE* is taken from Rasmussen et al. (2015)S30 in the Danish population; for estimates in different ethnicities, please refer to Raichlen and Alexander (2014).S31 The relative risks of amyloidosis and tauopathy are taken from Yu et al. (2019)S32 and Ebenau et al. (2020)S33; of neurodegeneration (neurofilament light) from Kern et al. (2019) S34 and de Wolf et al. (2020)S35; of subcortical cerebrovascular disease from Inzitari et al. (2009),S36 Kitagawa et al. (2015),S37 Sigurdsson et al. (2017),S38 and Inzitari et al. (2007)S39. The relative risk of amyloidosis and amyloidosis and neurodegeneration is computed from the 10-year risk reported in Brookmeyer & Abdalla (2018).^23^ The relative risks of genetic risk factors and potentially modifiable risk factors of brain pathology are generally adjusted for each other but communality with potentially modifiable risk factors without measured pathology is not taken into account.

b CAIDE: Cardiovascular Risk Factors, Aging and Dementia Score. ANU-ADRI: Australian National University Alzheimer's Disease Risk Index. BDSI: Brief Dementia Screening Indicator. All scales include age, CAIDE also sex and cholesterol, ANU-ADRI also sex, cognitive stimulating activities, and fish intake, and BDSI also difficulty with instrumental activities of daily living and previous stroke.

c The threshold for the definition of hypertension differ according to the monitoring device and setting.S9.

d Relative risks are reported for the 10-year risk of dementia in women, based on Brookmeyer & Abdalla (2018).^23^ Relative risks for men are only marginally different.

**Table 3:** The communication of the risk for dementia to cognitively healthy persons.

1	**Investigate risk perception and understanding**, i.e. educational attainment, intellectual abilities, personality traits (optimism versus pessimism), cognitive bias, mood, expectations, personal situation, preferences, values, risk-taking attitudes, numerical literacy including ability to understand numerical values and probability, preference for numerical format of risk figures, and initial beliefs about risk level including prior real-life experiences.S40
2	**Ask why's and what's**, i.e. why the person wants to know their risk of dementia, what the person's disease narratives and expectations are, and probe what they know about the pathophysiology and natural history of neurodegenerative diseases and their risk factors. Weigh the potential benefit and harm of disclosing the risk to the individual, the family, and the potential future caregiver, including the potential impact on employment and insurance, and expectations about the process of risk assessment and its actionability. Explore any reason for not wanting to know their dementia risk and take a shared decision on whether or not to continue with risk disclosure.
3	**Fill gaps of knowledge** with tailored information about the concept of risk, disease risk factors, and neurodegenerative diseases before deciding whether or not to continue the risk communication process.
4	**Use plain language**, i.e. present focused, well-structured, and logically sequenced information, and reduce or eliminate clinical and statistical jargon.
5	**Avoid use of qualitative risk descriptors**, e.g. “a high risk”, or “many people”.
6	**Present precise risk information**, such as frequencies “65 out of 100 individuals like you” or percentages, e.g. “65% of individuals similar to you”. When delivering this information, make sure to use estimates from large and representative cohorts where the key variables of age, gender, education, socio-economic status, and ethnicity are taken into account.
7	**Use familiar risk factors to benchmark dementia risk factors**, e.g. “the risk for dementia associated with having both amyloid and tau in the brain versus having none is of magnitude similar to the risk of death for lung cancer of smokers versus non-smokers”.
8	**Use mixed framing**, as order and framing affect risk perception. E.g.: “35 out of 100 individuals like you will develop dementia in 3 years' time [negative framing] and 65 out of 100 individuals like you will not develop dementia in 3 years' time [positive framing]”.
9	Use visual representation of risks, such as bar charts or icon arrays in addition to numerical risks (www.iconarray.com). E.g. use panel A when discussing the 10-year Alzheimer's dementia risk for a 75-year-old woman with neither amyloidosis nor neurodegeneration. The 2 blue and 98 black stick figures denote a risk of 2%.^23^
	AB
10	**Use an incremental risk format for interventions**, e.g. by displaying the risk with and without intervention in the same icon array. E.g. use panels A and B when discussing the 10-year Alzheimer's dementia risk for a 75-year-old woman with amyloidosis and neurodegeneration. The 2 blue stick figures denote the baseline risk (2%), the 19 red ones the incremental risk associated with amyloidosis and neurodegeneration, amounting to a global risk of 21%.^23^
11	**Draw attention to the risk time interval**, e.g. “this graph displays the risk in the next 5 years and this other the risk over your entire lifetime”
12	**Present absolute risks** instead of relative, e.g. “50% of people with one copy of the ε4 variant of APOE allele will develop dementia in their lifetime compared to 20% for people with no ε4”.
13	**Communicate APOE and amyloid risks with the same format as for lifestyle risks**, e.g. emphasize that APOE-ε4 and amyloid are neither necessary nor sufficient to develop cognitive impairment and dementia.
14	**Consider post-communication psychological support**. E.g. persons homozygotes for the ε4 allele of APOE, whose lifetime risk of developing Alzheimer's dementia is remarkably high.S41

References cited in the table can be found in the Supplementary material.

### Assessment of risk of cognitive decline

The assessment of the risk of unimpaired persons to develop cognitive impairment or dementia should build on a shared and transparent pathophysiological framework of neurodegenerative diseases and in particular Alzheimer's disease, by far the most frequent form of dementia. The conceptual framework underlying the current exercise builds on accepted risk factors for any dementia,^22^ and risk factors for the most frequent dementia type, Alzheimer's disease. The definition of Alzheimer's disease that we use is that of the International Working Group 2021 (panel 1) where biomarker positivity to brain amyloidosis and tauopathy in persons with no cognitive impairment denotes a risk condition.^25^ All risk factors are reported in Table 2 and categorized into genetic, potentially modifiable without measured brain pathology (in early life, midlife, and late life), and potentially modifiable of brain pathology. The magnitude of the risks, expressed in terms of relative risk, in an individual with multiple risk factors allows to stratify individuals into those with low, intermediate, and high risk, and prioritize interventions based on the ranking of relative risks.

The estimate of the global risk is a complex exercise requiring relative risks adjusted for frequently co-occurring risk factors (e.g. diabetes and obesity), effect of combination of risk factors, and population specific factors (e.g. genetics). The relative risks of potentially modifiable risk factors without measured brain pathology reported in Table 2 are from meta-analyses where studies usually adjusted for age, sex, and education as a minimum, with some studies adjusting for other risk factors of the same category. The relative risks of genetic and potentially modifiable risk factors of brain pathology are adjusted by age and sex, but none is adjusted for other risk factors and are thus likely to be over-estimated. This issue can be partly overcome by using dementia risk scales, that by design take communality of risk factors into account. However, dementia risk scales were developed in the pre-biomarker era and fail to take these into account, with the only exception of CAIDE (Cardiovascular Risk Factors, Aging and Dementia) that can optionally include the *APOE* genotype although this version has not been shown superior to the one without *APOE* genotype. Another significant limitation of available dementia risk scales is that they are limited to a relatively restricted age range (39–64 years for CAIDE and 65 years and older for ANU-ADRI—Australian National University Alzheimer's Disease Risk Index and BDSI—Brief Dementia Screening Indicator). Future longitudinal studies assessing simultaneously all risk factors reported in Table 2 and their association with brain pathology (amyloidosis, tauopathy, neurodegeneration, and subcortical cerebrovascular disease) in representative cohorts will allow more accurate estimates.

The assessment tool is a key issue when evaluating risk. Different assessment tools of variable accuracy are often available for the same risk factor (e.g. self-report and pure tone audiometry for hearing loss). Table 2 shows a suggested list of practical assessment tools, developed by the European Task Force for BHSs.^21^ In general, the more accurate tools have lower feasibility in a clinical setting and higher costs. It should be noted that the relative risks of potentially modifiable risk factors such as diabetes and obesity have been computed from meta-analyses of large epidemiological cohort studies, where risk factor assessment varied considerably (e.g. random glucose versus fasting glucose/HbA1C for diabetes diagnosis). The added value of using technologically more intensive albeit more accurate tools is likely to change the sensitivity and specificity of these biomarkers and might impact the estimate of the associated risk of incident dementia. Biomarkers of brain pathology allow the assessment of risk factors associated with moderate-to-high relative risks, but are currently resource intensive (MRI, lumbar puncture or PET). However, the validation of blood biomarkers is advancing fast and might soon complement or maybe replace the current ones.^26^^,^^27^

Risk assessment taking into account factors other than those potentially modifiable without measured brain pathology (frequently referred to as “lifestyle risk factors”) has a radical impact on risk communication and the planning of interventions for personalized prevention of cognitive decline. Lifestyle risk factors have low relative risks, ranging between 1.1 and 1.9 (Table 2) and clinical advice can be reasonably given based on their presence/absence. Genetic risk factors and those of brain pathology have much higher relative risks, generally in the 2–10 range and occasionally above 20 (Table 2). This imposes a ranking of all the risk factors of a given individual, raises challenges of communicating risks of different size, and requires prioritizing risk reduction interventions based on the ranking.

We acknowledge that available estimates of relative risk are imperfect as they come from clinically oriented and volunteer cohort studies, and these have not addressed the interaction of genetic risk factors with potentially modifiable risk factors and brain pathology. We therefore cannot accurately compute relative risks for dementia if, for example, hearing loss, amyloidosis, and neurodegeneration are present in the same person, nor can we determine how much of brain pathology can be attributed to modifiable risk factors such as hypertension or diabetes themselves. However, we believe that currently available estimates (Table 2), albeit imperfect, provide actionable information.

The relative risk of dementia associated with markers of brain pathology is higher at a younger age: the computed 10-year risk of the combination of amyloidosis and neurodegeneration at 60 years of age is 21.4 and 25.3 in women and men and just 4.3 and 5.5 in women and men at age 85 (computed from Brookmeyer & Abdalla, 2018^23^). This is likely to be due to competing mortality and possibly cognitive reserve. Someone in their 80's who has survived to that age and has not developed dementia despite brain pathology is likely to die before developing dementia, based on average life expectancies. The fact that they have not developed dementia by this age but have measurable brain pathology also may indicate some sort of resilience against cognitive aging or cognitive reserve. Future large-scale population-based studies will need to measure all risk factors together and refine risk estimates by taking communalities and interactions into account.

### Risk communication

Effective communication of dementia risk is paramount for shared decision making about whether or not to engage in interventions aimed at risk reduction. Risk disclosure is a medical act by itself, because of its potential impact on psychological and mental health of the individual.^24^ The continuous nature of risk makes risk communication an exercise more complex than disclosing the dichotomous event of a diagnosis (affected/not affected). The general population, and even health professionals, often do not correctly interpret probabilities and epidemiological data.^28^

Effective risk communication is affected by individual characteristics (Table 3). The decision to communicate about the individual's dementia risk should be a shared decision between the health care professional and the person potentially at risk. In the field of clinical neuroscience, structured risk communication protocols are available for transmissible genetic conditions such as Huntington's and autosomal dominant Alzheimer's disease.^29^^,^^30^ A useful starting point for the communication of dementia risk are the protocols developed for individual risk factors such as APOE and brain amyloidosis.^31^, ^32^, ^33^ The following recommendations have been developed based on a review of the literature and personal experience of the coauthors of the present manuscript.^24^

Table 3 reports concrete examples that will help healthcare professionals to use a language appropriate to the person's knowledge background and deliver the concept of risk in a way that is meaningful to the individual.

### Interventions for personalised prevention of cognitive decline

Interventions are feasible and may be effective for the potentially modifiable risk factors other than specific testable brain pathology (amyloidosis, tauopathy, and neurodegeneration). Evidence in persons at high risk for dementia shows that simultaneous multi-domain interventions on cognition (e.g. with computerized games), physical fitness (e.g. with muscle strength and aerobic exercise), nutrition (e.g. with nutritional education or supplementation), and vascular risk factors (e.g. with strict control of blood pressure and diabetes) carried out over a sufficiently long term (e.g. 2 years) might slow age-associated cognitive decline.^8^ Such programmes are generally well accepted by those recruited to trials; however, these may not be representative of those most at risk of dementia in society in general, and programmes have implications that are resource intensive both for them and care providers.

Preliminary evidence from the FINGER and MAPT trials indicates that such multi-domain interventions might be particularly effective in persons at higher risk due to *APOE*-ε4 carrier status,^34^ shorter leukocyte telomere length,^35^ brain beta-amyloidosis,^12^ and higher CAIDE score,^13^ suggesting the possibility of precision risk reduction based on genetic, biological, or clinical features. These are *post-hoc* analyses and caution in their interpretation is necessary, ideally to be tested *a priori* in further studies. Other findings including greater benefit in persons with greater brain cortical thickness,^36^ no history of cardiovascular disease^37^ or untreated hypertension^37^ suggest that there is room to better define target populations for precision risk reduction. High risk and high prevention potential groups are not necessarily identical, and further studies are needed to define the optimal window of opportunity for risk reduction at individual level.

Pharmacologic interventions may become available to persons with potentially modifiable risk factors of brain pathology, e.g. the monoclonal antibodies currently under testing in patients with cognitive impairment due to AD pathology (aducanumab, donanemab, and lecanemab).^38^, ^39^, ^40^ Further in the future, gene therapies targeting APOE4, APOE2, and NGF in addition to beta-amyloid and tau may add to the pharmacological *armamentarium*.^41^ Importantly, as of today evidence is lacking on risk reduction for incident cognitive impairment and dementia by these or other drugs in persons with no cognitive impairment.

At the time of the writing of this article (first quarter 2022), 14 preventive clinical trials are ongoing in cognitively unimpaired participants at high risk of sporadic Alzheimer's dementia; five use anti-amyloid immunotherapies, three omega-3 fatty acids, two drugs active on glucose metabolism, one an anti-tau antibody, one a blood pressure lowering drug, one memantine, and one orexin antagonist (personal data). If these trials show a beneficial effect, exciting new avenues might open up for precision risk reduction based on molecular profile. The availability of anti-tau drugs and drugs active on inflammatory and other pathways^42^ might in the coming years expand the pharmacologic armamentarium and pave the way towards pharmacologic intervention on multiple risk factors as we currently do for vascular prevention. A clinical trial will soon test the preventive effect of the association of anti-amyloid and anti-tau monoclonal antibodies in persons at risk for the rare autosomal dominant form of AD.^43^ Combination trials for the much more frequent sporadic form may follow suit.

### Interventions for cognitive enhancement

People who currently come to memory clinics seeking risk assessment and risk reduction often also ask for interventions for improving cognitive function. Interventions that have a sufficiently large body of evidence focus on cognitive and physical training. NIBS is reviewed for its potential despite a limited number of studies currently being available.

A number of studies have investigated the efficacy of repeated practice, physical training, and strategic learning in persons with subjective cognitive decline (Supplementary Table). Most studies found some degree of efficacy on outcomes that the intervention was supposed to target (subjective memory, objective memory, executive functions and attention, and meta-memory) while evidence in favor of efficacy was more mixed for non-trained cognitive domains and functions (global cognition, proximal and distant transfer, activities of daily living, mood and quality of life, and motivation). Evidence was particularly suggestive in favour of a beneficial effect of cognitive intervention (especially strategic learning) on meta-memory, or the introspective knowledge of one's own memory capabilities.

There is limited evidence base on NIBS. A GRADE analysis of NIBS studies was not performed for our original report on cognitive enhancement^44^ and was done specifically for the current review. While the overall quality of evidence was rated as low (Supplementary Table), the preliminary results on potentially beneficial effects of NIBS on cognitive performance are encouraging and call for further research in this field.

Cognition enhancing drugs or nootropics have thus far proven disappointing when it comes to consistently showing efficacy in rigorous double-blind placebo-controlled trials. A search in the Cochrane collaboration database retrieves 3 reviews of nootropics in persons with normal cognitive performance. One on dehydroepiandrosterone (DHEA) supplementation for cognitive function in healthy elderly people concludes against any evidence of a beneficial effect of DHEA supplementation on cognitive function of dementia free middle-aged or elderly people.^45^ Another on ginseng for cognition concluded that despite a lack of convincing evidence to show a cognitive enhancing effect of *Panax ginseng* in healthy participants, all the 5 selected studies suggested improvement of some aspects of cognitive function, behavior and quality of life.^46^ A review on L-carnitine for cognitive enhancement in people without cognitive impairment found only 2 trials satisfying methodological standards, but in both cases evidence was of very low quality, and the authors were unable to draw any conclusions about the effect of the drug.^47^

## Cautionary notes

We have outlined the instruments and activities of next-generation memory clinics (so-called BHSs) with the mission to prevent cognitive impairment and dementia and improve the wellbeing and cognitive performance of individuals with subjective cognitive decline and worried well persons. We have provided recommendations based on current best evidence on the cornerstone interventions of BHSs, namely how to assess risk for cognitive impairment and dementia, how to communicate about it, interventions for risk reduction, and interventions for cognitive enhancement. We emphasize that evidence on the clinical usefulness of the individual components is of variable quality and that the efficacy of BHSs as a global package remains to be demonstrated.

This section highlights issues that should be taken into account when setting up a pilot BHS, such as the geographical scope and generalizability of the concept, the patient population, ethical and legal issues, and gaps of scientific evidence.

### Geographical scope and generalizability

The geographical scope of our initiative is restricted to Europe, where BHSs might be leveraging on the structure and function of current memory clinics, whose main features are summarized in Table 1, depending on local opportunities and resources. Indeed, the core structure of current memory clinics comprising medical specialists with specific expertise in cognitive impairment and dementia, psychologists, and various therapists as well as a technical MR imaging platform is remarkably similar all over Europe.^48^ More variable is access to and use of imaging (PET, SPECT) and fluid (CSF) biomarkers, generally more extensive in academic memory clinics.^49^^,^^50^ Similarly, BHSs can be envisioned with variable access to and use of imaging and fluid biomarkers for risk assessment and stratification.

It should be emphasized that the four cornerstone interventions mentioned above and represented in Fig. 1 should not be regarded as a rigid recipe that can be generalized to any BHSs in Europe right away. Local circumstances will significantly affect the viability and operations of BHSs such as availability of expertise/facilities, health policies, resource opportunities/restrictions, and reimbursement policies.

### Target population

The target population of BHSs are individuals with subjective cognitive decline and worried-well persons. Population screening will not be a mandate of BHSs. It can be anticipated that some of those persons with subjective cognitive decline or worried-well seeking help in BHSs will have functional cognitive disorders.^51^ Others may have potentially treatable conditions such as mental distress, polypharmacy, intracranial mass, perimenopause, suboptimal sleep or sleep-apnea syndrome.^52^ These cases will need to be identified and directed to the appropriate specialists before engaging in a dementia prevention exercise along the lines spelled out in this paper. Likewise, for a proportion of individuals, clear information about the functioning of their brain and expected age-associated changes may help alleviate concerns related to their cognitive functioning.

### Ethical and legal framework

Pilot BHSs should ideally rely on the ethical and legal framework of health service research projects with the collection of patient data to validate the facilities *per se*, including *ad hoc* ethical clearance, and possibly informed patient consent. In many European countries, financial resources for pilot BHSs would come from research rather than from current care funds, thus not taking resources away from existing memory clinics and cognitively impaired patients. Non-validated interventions should ideally be implemented in the context of research interventions on human beings and undergo the usual ethical review and informed consent process. In some legal contexts, they could be implemented as non-validated diagnostics and therapeutics representing best clinical effort in the absence of data, where ethics review and informed consent would still be required. In all cases, careful adaptation and compliance with national legislation is recommended.

### Gaps of scientific evidence

We acknowledge that a number of gaps of scientific evidence need to be addressed before BHSs can take off beyond pilot experiences and move into production mode. The clinical validity of blood-based biomarkers of neurodegeneration and Alzheimer's molecular pathology and polygenic risk scores^53^^,^^54^ will need to be completed with thresholds denoting incremental risk.^26^ The predictive value of digital biomarkers and the incremental predictive value over blood-based biomarkers will need to be evaluated. Biomarker status disclosure raises ethical issues which may be resolved in part by the accruing biomarker data but will require substantial cultural and psychosocial investigation.^55^ Large population-based longitudinal observational studies will provide estimates of the risk associated with different combinations of genetic and potentially modifiable risk factors by adjusting for communality and accounting for interactions of risk factors, thus allowing to estimate the overall dementia risk of an individual, allocate them in risk strata, and develop stratum-specific risk reduction interventions. These studies will need to involve underrepresented and disadvantaged communities to guarantee generalizability. Large-scale education programs will result in increased awareness of the general population on risk factors for cognitive health, ad-hoc studies will develop and implement brain health-specific communication strategies on an individual level, and the diffusion of online brain health registry will facilitate the participation of citizens in innovative preventive interventions.^56^^,^^57^ Training courses will need to be created on dementia risk communication.

The generalizability and efficiency of multi-domain interventions needs to be verified in multiple geographically and culturally diverse settings,^58^ more evidence should be collected on their dose-effect relationship,^59^ and the differential sensitivity to intervention of specific genetic, biological, and clinical subgroups will need to be clarified. Drugs targeting the core pathologies of AD (anti-amyloid, anti-tau, and secretase inhibitors) and non-conventional interventions (e.g. probiotics and microbiome-based drugs, metabolism and bioenergetics, photo-oxygenation, SV2A modulators, active on mitochondrial stress, transfusion with young blood, among others)^60^^,^^61^ will need to show efficacy at reducing the risk for cognitive impairment and dementia.

Even at a time where the prevention of cognitive impairment and dementia is routine, the request for cognitive enhancement will likely stay on, and may even increase. Non-pharmacological interventions for cognitive enhancement will need to provide ultimate proof of efficacy, possibly by combining different approaches into multi-domain non-lifestyle interventions (e.g. NIBS, nutritional supplementation, and cognitive training). Patient preferences on BHS interventions will need to be taken into account^62^ as well as the interaction of BHSs with more integrative approaches for optimizing intrinsic capacity and functional ability in healthy ageing such as WHO's ICOPE (Integrated Care for Older People).^63^

## The roadmap for the validation of BHS

We propose a roadmap for the validation of BHSs that may pave the way to wider deployment in clinical practice (Fig. 2). Pilot BHSs should implement all four cornerstone interventions of Fig. 1 but might place different emphasis on each according to local availability of expertise and technical facilities. Pilot BHSs will need new competences to deliver risk assessment, risk communication, personalised prevention interventions, and cognitive enhancement. However, the expertise of current memory clinics in terms of the pathophysiology of cognitive disorders and neuropsychological assessment as well as the technological platform for genetic, imaging, and fluid biomarker assessment might usefully feed into BHSs.

Local policy-makers would need to consider the contribution to dementia reduction for their populations that local BHSs might make, and at what cost. Depending on local opportunities and restrictions, BHSs may be set up as clinical spinoffs of currently active memory clinics, as brand new services with their own personnel and technological platform,^17^^,^^64^ or develop as a specific health care offering within current memory clinics. Business models and the involvement of private or public payers may vary according to local reimbursement policies and as evidence accrues on the effectiveness of monoclonal antibodies and other molecules for prevention in cognitively unimpaired persons at risk for dementia. Outcome evaluation and research including a longitudinal patient registry should be embedded in any newly developed BHS to evaluate costs and effectiveness and dynamically revise the health offer based on cost-effectiveness data. Effectiveness should be based on pre-defined endpoints such as individual dementia risk reduction in the short-term and decreased incidence of dementia and cognitive impairment of the BHS-treated population in the long-term. Pilot experiences should be coordinated within and between European countries.

In the long term, the question of who should be in charge of cognitive impairment and dementia prevention, whether the specialist or the general practitioner, will likely be a contentious point in most health systems in Europe. It is likely that elderly citizens themselves will be actively engaged in these clinical decisions.^65^^,^^66^

We believe that the delivery of cognitive impairment prevention will evolve dynamically and follow scientific and technological developments. As long as accurate risk profiling requires genotyping, PET scans, and CSF biomarkers, specialists will most likely be the key actors. When the predictive power of blood-based and digital biomarkers is proven to be sufficiently valuable, and their technological platform(s) become commonplace as is the case of cholesterol testing, at least the first level of risk assessment may be delegated to GPs. Some of the other variables that will drive the integration of GPs and specialists into prevention on a large scale will be: the availability of drugs with a demonstrated preventive efficacy, their tolerability profile, their cost, and the need for theranostics. Knowledge on risk factors for non-Alzheimer's neurodegenerative dementias and specific risk reduction interventions, currently extremely limited, may be factored-in as more evidence accrues.

In any case, BHSs will need to strike a balance between an individualized and population focus for public health prevention to meet ethical standards of justice and health equity and take into account socioeconomic factors, including cultural differences, regional heterogeneity, health system structures, socioeconomic levels, and disparities in social determinants of health.^67^ The model may be followed of the Scottish BHSs initiative, where services were co-designed with marginalized communities, and mobile/roving brain health services will be provided as well as digital and telehealth services.^5^ Active evaluation of the cost-effectiveness and burden-benefit ratios of the services will need place specific emphasis on disadvantaged and underrepresented groups.^5^ We believe that the greatest societal benefits to cognitive health will be provided by a dynamically coordinated synergy of population-wide risk reduction strategies with individualized interventions such as BHSs.^68^

## Contributors

GBF was the initiator of the BHS initiative together with BD, PS, CR, and JFD. An online workshop took place on June 15 and 16, 2020, where DA, MBA, ABG, RM, CM, JLM, JR, AS, RS, and LNCV led the working groups that led to the publication of the *Alzheimer's Research and Therapy* series of articles, and CB, GC, GBF, MK, DJL, JLM, WvdF, and JFD were their supervisors. The executive board of the June 15-16 2020 workshop consisted of GBF, DA, FR, and JFD. All other coauthors except NM, JLC, and OGR were active participants to the workshop. NM, JLC, and OGR were involved in the writing of the current paper and provided *ad hoc* expertise. GBF drafted the first version of the current paper and revisions of drafts. All authors contributed to sections of the paper, and all revised it for important intellectual content.

## Role of the funding source

The workshop of June 15-16 2020 was funded in part by the 10.13039/100000001Swiss National Science Foundation (SNSF) under grant agreement IZSEZ0_193593. The SNSF had no role in the development of the concepts and ideas illustrated in the present manuscript nor in its writing and in the decision to submit the paper for publication. None of the coauthors received any compensation related to the workshop or the writing of the scientific manuscripts that ensued, including the present one. All authors had full access to the data in the study, and they accept responsibility to submit for publication.

## Declaration of interests

GBF has received funding through the Private Foundation of 10.13039/501100006388Geneva University Hospitals from: A.P.R.A.—Association Suisse pour la Recherche sur la Maladie d’Alzheimer, Genève; 10.13039/501100023259Fondation Segré, Genève; Race Against Dementia Foundation, London, UK; Fondation Child Care, Genève; Fondation Edmond J. Safra, Genève; Fondation Minkoff, Genève; Fondazione Agusta, Lugano; McCall Macbain Foundation, Canada; Nicole et René Keller, Genève; Fondation AETAS, Genève. He has received funding through the 10.13039/501100006389University of Geneva or Geneva University Hospitals: for IISSs from ROCHE Pharmaceuticals, OM Pharma, EISAI Pharmaceuticals, Biogen Pharmaceuticals, and Novo Nordisk; for competitive research projects from: H2020, 10.13039/501100010767Innovative Medicines Initiative (IMI), IMI2, 10.13039/100000001Swiss National Science Foundation, and VELUX Foundation; for consulting from: Biogen, Diadem, Novo Nordisk, and Roche; for honoraria for lectures, presentations, speakers bureaus, manuscript writing, or educational events from: Biogen, Roche, Novo Nordisk, and GE HealthCare.

DA received funding by the Fondation Recherche Alzheimer and the 10.13039/100000001Swiss National Science Foundation (project CRSK-3_196354/1).

FR is funded in part by the 10.13039/100000001Swiss National Science Foundation under grant agreement 320030_182772: Brain connectivity and metacognition in persons with subjective cognitive decline (COSCODE).

NV received research support from Fondation Bettencourt-Schueller, Fondation Servier, Union Nationale pour les Intérêts de la Médecine (UNIM) and Fondation pour la Recherche sur l’Alzheimer; travel grant from the Movement Disorders Society, Merz-Pharma and GE Healthcare SAS; is an unpaid sub-investigator in NCT04241068 (aducanumab, Biogen), NCT04437511 (donanemab, Eli-Lilly), NCT04592341 (gantenerumab, Roche), NCT03887455 (lecanemab, Eisai), NCT03352557 (gosuranemab, Biogen), NCT03828747 and NCT03289143 (semorinemab, Roche), NCT04619420 (JNJ-63733657, Janssen–Johnson & Johnson), NCT04374136 (AL001, Alector), NCT04592874 (AL002, Alector); and has given unpaid lectures in symposia organized by Eisai.

FB is supported by the NIHR biomedical research centre at UCLH. FB is a steering committee or iDMC member for Biogen, Merck, Roche, EISAI. Consultant for Roche, Biogen, Merck, IXICO, Jansen, Combinostics. Research agreements with Novartis, Merck, Biogen, GE, Roche. FB is co-founder and share–holder of Queen Square Analytics LTD.

KB has served as a consultant, at advisory boards, or at data monitoring committees for Abcam, Axon, BioArctic, Biogen, JOMDD/Shimadzu. Julius Clinical, Lilly, MagQu, Novartis, Ono Pharma, Pharmatrophix, Prothena, Roche Diagnostics, and Siemens Healthineers, and is a co-founder of Brain Biomarker Solutions in Gothenburg AB (BBS), which is a part of the GU Ventures Incubator Program, all outside the work presented in this paper.

GC has received research support from the 10.13039/501100007601European Commission Horizon 2020 research and innovation programme (grant agreement number 667696), Fondation d'entreprise MMA des Entrepreneurs du Futur, Fondation Alzheimer, Programme Hospitalier de Recherche Clinique, 10.13039/501100001665Agence Nationale de la Recherche, Région Normandie, 10.13039/501100003750Association France Alzheimer et maladies apparentées, 10.13039/100014808Fondation Vaincre Alzheimer, Fondation Recherche Alzheimer and Fondation pour la Recherche Médicale (all to Inserm), and personal fees from Inserm, Fondation Alzheimer and Fondation d'entreprise MMA des Entrepreneurs du Futur.

JG has received funding for the EURO-FINGERS project which is supported through the following funding organisations under the aegis of JPND www.jpnd.eu: Finland, Academy of Finland; Germany, Federal Ministry of Education and Research; Spain, National Institute of Health Carlos III; Luxembourg, National Research Fund; Hungary, National Research, Development and Innovation Office; Netherlands, Netherlands Organisation for Health Research and Development; Sweden, 10.13039/501100004359Swedish Research Council. Grant agreement: INTER/JPND/19/BM/14012609.

AN reports grants from 10.13039/501100000304Parkinson's UK, 10.13039/100015652Barts Charity, Cure Parkinson's, NIHR, 10.13039/501100006041Innovate UK, Virginia Keiley benefaction, Alchemab, Aligning Science Across Parkinson's and Michael J Fox Foundation. Consultancy and personal fees from Astra Zeneca, AbbVie, Profile, Roche, Biogen, UCB, Bial, Charco Neurotech, uMedeor and Britannia, outside the submitted work.

JMR and DJL are funded by Alzheimer's Research UK and the 10.13039/100012338Alan Turing Institute/Engineering and Physical Sciences Research Council (EP/N510129/1). DJL is also funded by National Institute for Health Research (NIHR) Applied Research Collaboration (ARC) South West Peninsula, 10.13039/501100000925National Health and Medical Research Council (NHMRC), 10.13039/100000049National Institute on Aging/10.13039/100000002National Institutes of Health (RF1AG055654).

WvdF has been funded by ZonMW, NWO, EU-FP7, EU-JPND, 10.13039/501100010969Alzheimer Nederland, Hersenstichting CardioVascular Onderzoek Nederland, Health Holland, Topsector Life Sciences & Health, stichting Dioraphte, Gieskes-Strijbis fonds, stichting Equilibrio, Edwin Bouw fonds, Pasman stichting, stichting Alzheimer & Neuropsychiatrie Foundation, Philips, Biogen MA Inc, Novartis-NL, Life-MI, AVID, Roche BV, Fujifilm, Combinostics. WvdF holds the Pasman chair. WvdF, PS and LNV are recipients of ABOARD, which is a public-private partnership receiving funding from ZonMW (#73305095007) and Health ∼ Holland, Topsector Life Sciences & Health (PPP-allowance; #LSHM20106). WvdF, PS, and LNV are recipients of the EU Joint Programme - Neurodegenerative Disease Research (JPND) project EURO-FINGERS (ZonMW-Memorabel #733051102). WvdF has performed contract research for Biogen MA Inc, and Boehringer Ingelheim. WvdF has been an invited speaker at Boehringer Ingelheim, Biogen MA Inc, Danone, Eisai, WebMD Neurology (Medscape), Swiss National Science Foundation, Switzerland, European Commission Horizon 2020, Belgium, Springer Healthcare. WvdF is consultant to Oxford Health Policy Forum CIC, Roche, and Biogen MA Inc. WvdF participated in advisory boards of Biogen MA Inc and Roche. All funding is paid to her institution. WvdF was associate editor of Alzheimer, Research & Therapy in 2020/2021. WvdF is associate editor at Brain.

BV is an investigator in clinical trials sponsored by Biogen, Lilly, Roche, Eisai Pharmaceuticals, and the Toulouse University Hospital (Inspire Geroscience Program). He has served as scientific advisor for Biogen, Alzheon, Green Valley, Norvo Nordisk, Longeveron, but received no personal compensation. He has served as consultant and/or scientific advisor for Roche, Lilly, Eisai, TauX with personal compensation.

All other coauthors declare no competing interests.
