# Supplementary material for: Dementia prevention in memory clinics: recommendations from the European task force for brain health services
Source: Lancet Reg Health Eur. 2023 Jan 31;26:100576. doi: 10.1016/j.lanepe.2022.100576 (PMC9989648; doi:10.1016/j.lanepe.2022.100576)
Supplement: Supplementary material [file mmc1.docx]

**Dementia prevention in the memory clinic of the future: Recommendations from the European Task Force for Brain Health Services**

Giovanni B. Frisoni,^a^ Daniele Altomare,^a^ Federica Ribaldi,^a^ Nicolas Villain,^b,c^ Carol Brayne, Naaheed Mukadam, Marc Abramowicz, Frederik Barkhof, Marcelo Berthier, Melanie Bieler-Aeschlimann, Kaj Blennow, Andrea Brioschi Guevara, Emmanuel Carrera, Gael Chételat, Chantal Csajka, Jean-François Demonet, Alessandra Dodich, Valentina Garibotto, Jean Georges, Samia Hurst, Frank Jessen, Miia Kivipelto, David J. Llewellyn, Laura McWhirter, Richard Milne, Carolina Minguillón, Carlo Miniussi, José Luis Molinuevo, Peter M Nilsson, Alastair Noyce, Janice Ranson, Oriol Grau-Rivera, Jonathan M. Schott, Alina Solomon, Ruth Stephen, Wiesje van der Flier, Cornelia van Duijn, Bruno Vellas, Leonie N.C. Visser, Jeffrey Cummings, Philip Scheltens, Craig Ritchie, Bruno Dubois

*The Lancet Regional Health - Europe*

**Table of contents**

Supplementary table page 2

Supplementary references page 3

**Supplementary table**. Randomized controlled studies of training interventions for cognitive enhancement in persons with subjective cognitive decline. Each dot represents a study where interventions were repeated practice, physical training, or strategic learning. Outcomes of the intervention were specific cognitive functions, global cognition, and non-trained domains. The intensity of the color denotes the quality of the evidence according to the GRADE evaluation system of healthcare (Grading of Recommendations Assessment, Development and Evaluation), where green denotes studies supporting efficacy (better outcomes in treated than untreated), pink denotes studies not supporting efficacy (similar outcomes in treated and untreated), and white denotes studies not studying the outcome. Light shades of green and pink denote low quality of the evidence according to GRADE, the darker shades moderate quality, and the darkest shades (green only) high quality. Proximal transfer: extension of the benefit to a non-trained sub-domain of the same cognitive function (training of e.g. working memory, a subdomain of executive functions, benefits a non-trained sub-domain of executive functions e.g. mental flexibility). Distal transfer: extension of the benefit to a non-trained cognitive function (training of e.g. executive functions benefits e.g. memory). Generalization to ADL: a form of distal transfer where benefit extends to activities of daily living.

|  | **Cognitive outcomes** | | | | **Non-trained domains and functions** | | | | | | | | |
| --- | --- | --- | --- | --- | --- | --- | --- | --- | --- | --- | --- | --- | --- |
|  | **Subjective memory** | **Objective memory** | **Executive functions and attention** | **Meta-memory** | **Global cognition** | **Proximal and distant transfer** | | **Generali- zation to ADL** | | **Mood and quality of life** | | **Moti-**  **vation** | |
| **Training interventions** |  |  |  |  |  |  | |  | |  | |  | |
| Repeated practice (7 studies^S42-S48^) |  |  |  |  |  |  | |  | |  | |  | |
| Physical training (4 studies^S49-S52^) |  |  |  |  |  |  | |  | |  | |  | |
| Strategic learning (11 studies^S53-S63^) |  |  |  |  |  |  | |  | |  | |  | |
| **Non-invasive brain stimulation** |  |  |  |  |  |  | |  | |  | |  | |
| Transcranial electric or magnetic stimulation (3 studies^8,S64,S65^) |  |  |  |  |  |  |  | |  | |  | |  |

**Supplementary references**

(to Table 2 and Table 3 in the main paper, and Supplementary table)

S1 Kivipelto M, Ngandu T, Laatikainen T, Winblad B, Soininen H, Tuomilehto J. Risk score for the prediction of dementia risk in 20 years among middle aged people: a longitudinal, population-based study. *Lancet Neurol.* 2006; **5**: 735–41.

S2 Anstey KJ, Cherbuin N, Herath PM. Development of a New Method for Assessing Global Risk of Alzheimer’s Disease for Use in Population Health Approaches to Prevention. *Prev Sci*. 2013; **14**: 411–21.

S3 Anstey KJ, Cherbuin N, Herath PM, et al. A self-report risk index to predict occurrence of dementia in three independent cohorts of older adults: The ANU-ADRI. *PLoS One*. 2014; **9**. DOI:10.1371/journal.pone.0086141.

S4 Barnes DE, Beiser AS, Lee A, et al. Development and validation of a brief dementia screening indicator for primary care. *Alzheimer’s Dement*. 2014; **10**: 656-665.e1.

S5 Sharp ES, Gatz M. Relationship between education and dementia: an updated systematic review. *Alzheimer Dis Assoc Disord*. 2011; **25**: 289–304.

S6 Fagerlin A, Zikmund-Fisher BJ, Ubel PA. Helping patients decide: Ten steps to better risk communication. *J Natl Cancer Inst*. 2011; **103**: 1436–43.

S7 Pirozzo S, Papinczak T, Glasziou P. Whispered voice test for screening for hearing impairment in adults and children: systematic review. *BMJ*. 2003; **327**: 967–70.

S8 Corrigan JD, Bogner J. Initial reliability and validity of the Ohio State University TBI Identification Method. *J Head Trauma Rehabil*. 2007; **22**: 318–29.

S9 Williams B, Mancia G, Spiering W, et al. 2018 ESC/ESH Guidelines for the management of arterial hypertension. *Eur Heart J*. 2018; **39**: 3021–104.

S10 McKenna H, Treanor C, O’Reilly D, Donnelly M. Evaluation of the psychometric properties of self-reported measures of alcohol consumption: a COSMIN systematic review. *Subst Abuse Treat Prev Policy*. 2018; **13**. DOI:10.1186/S13011-018-0143-8.

S11 Razi SM, Manish G, Keshav GK, Sukriti K, Gupta A. Site or Size of Waist Circumference, Which one is more important in metabolic syndrome? *Int J Med Public Heal*. 2016; **6**: 69–72.

S12 Levis B, Sun Y, He C, et al. Accuracy of the PHQ-2 Alone and in Combination with the PHQ-9 for Screening to Detect Major Depression: Systematic Review and Meta-analysis. JAMA - J. Am. Med. Assoc. 2020; 323: 2290–300.

S13 Snaith RP. The Hospital Anxiety And Depression Scale. *Health Qual Life Outcomes*. 2003; **1**. DOI:10.1186/1477-7525-1-29.

S14 Lubben JE. Assessing social networks among elderly populations. *Fam Community Heal*. 1988; **11**: 42–52.

S15 Koenig HG, Westlund RE, George LK, Hughes DC, Blazer DG, Hybels C. Abbreviating the Duke Social Support Index for use in chronically ill elderly individuals. *Psychosomatics*. 1993; **34**: 61–9.

S16 Sylvia LG, Bernstein EE, Hubbard JL, Keating L, Anderson EJ. Practical guide to measuring physical activity. *J Acad Nutr Diet*. 2014; **114**: 199–208.

S17 Cosentino F, Grant PJ, Aboyans V, et al. 2019 ESC Guidelines on diabetes, pre-diabetes, and cardiovascular diseases developed in collaboration with the EASD. *Eur Heart J*. 2020; **41**: 255–323.

S18 Crous-Bou M, Gascon M, Gispert JD, et al. Impact of urban environmental exposures on cognitive performance and brain structure of healthy individuals at risk for Alzheimer’s dementia. *Environ Int* 2020; **138**. DOI:10.1016/J.ENVINT.2020.105546.

S19 Garibotto V, Boccardi M, Chiti A, Frisoni GB. Molecular imaging and fluid biomarkers of Alzheimer’s disease neuropathology: an opportunity for integrated diagnostics. *Eur J Nucl Med Mol Imaging*. 2021; **48**: 2067–9.

S20 Leuzy A, Ashton NJ, Mattsson-Carlgren N, et al. 2020 update on the clinical validity of cerebrospinal fluid amyloid, tau, and phospho-tau as biomarkers for Alzheimer’s disease in the context of a structured 5-phase development framework. *Eur J Nucl Med Mol Imaging*. 2021; **48**: 2121–39.

S21 Chiotis K, Saint-Aubert L, Boccardi M, et al. Clinical validity of increased cortical uptake of amyloid ligands on PET as a biomarker for Alzheimer’s disease in the context of a structured 5-phase development framework. *Neurobiol Aging*. 2017; **52**: 214–27.

S22 Hanseeuw BJ, Malotaux V, Dricot L, et al. Defining a Centiloid scale threshold predicting long-term progression to dementia in patients attending the memory clinic: an [ 18 F] flutemetamol amyloid PET study. *Eur J Nucl Med Mol Imaging*. 2021; **48**: 302–10.

S23 van der Kall LM, Truong T, Burnham SC, et al. Association of β-Amyloid Level, Clinical Progression, and Longitudinal Cognitive Change in Normal Older Individuals. *Neurology*. 2021; **96**: e662–70.

S24 Wolters EE, Dodich A, Boccardi M, et al. Clinical validity of increased cortical uptake of [ 18 F]flortaucipir on PET as a biomarker for Alzheimer’s disease in the context of a structured 5-phase biomarker development framework. *Eur J Nucl Med Mol Imaging*. 2021; **48**: 2097–109.

S25 Bischof GN, Dodich A, Boccardi M, et al. Clinical validity of second-generation tau PET tracers as biomarkers for Alzheimer’s disease in the context of a structured 5-phase development framework. *Eur J Nucl Med Mol Imaging*. 2021; **48**: 2110–20.

S26 Mielke MM, Syrjanen JA, Blennow K, et al. Plasma and CSF neurofilament light: Relation to longitudinal neuroimaging and cognitive measures. *Neurology*. 2019; **93**: E252–60.

S27 Cotta Ramusino M, Altomare D, Bacchin R, et al. Medial temporal lobe atrophy and posterior atrophy scales normative values. *NeuroImage Clin*. 2019; **24**. DOI:10.1016/J.NICL.2019.101936.

S28 Ribaldi F, Altomare D, Jovicich J, et al. Accuracy and reproducibility of automated white matter hyperintensities segmentation with lesion segmentation tool: A European multi-site 3T study. *Magn Reson Imaging*. 2021; **76**: 108–15.

S29 Rhodius-Meester HFM, Benedictus MR, Wattjes MP, et al. MRI Visual Ratings of Brain Atrophy and White Matter Hyperintensities across the Spectrum of Cognitive Decline Are Differently Affected by Age and Diagnosis. *Front Aging Neurosci*. 2017; **9**. DOI:10.3389/FNAGI.2017.00117.

S30 Rasmussen KL, Tybjærg-Hansen A, Nordestgaard BG, Frikke-Schmidt R. Plasma levels of apolipoprotein E and risk of dementia in the general population. *Ann Neurol*. 2015; **77**: 301–11.

S31 Raichlen DA, Alexander GE. Exercise, APOE genotype, and the evolution of the human lifespan. *Trends Neurosci*. 2014; **37**: 247–55.

S32 Yu JT, Li JQ, Suckling J, et al. Frequency and longitudinal clinical outcomes of Alzheimer’s AT(N) biomarker profiles: A longitudinal study. *Alzheimers Dement*. 2019; **15**: 1208–17.

S33 Ebenau JL, Timmers T, Wesselman LMP, et al. ATN classification and clinical progression in subjective cognitive decline. *Neurology*. 2020; 10.1212/WNL.0000000000009724.

S34 Kern S, Syrjanen JA, Blennow K, et al. Association of Cerebrospinal Fluid Neurofilament Light Protein With Risk of Mild Cognitive Impairment Among Individuals Without Cognitive Impairment. *JAMA Neurol*. 2019; **76**: 187–93.

S35 de Wolf F, Ghanbari M, Licher S, et al. Plasma tau, neurofilament light chain and amyloid-β levels and risk of dementia; a population-based cohort study. *Brain*. 2020; **143**: 1220–32.

S36 Inzitari D, Pracucci G, Poggesi A, et al. Changes in white matter as determinant of global functional decline in older independent outpatients: three year follow-up of LADIS (leukoaraiosis and disability) study cohort. *BMJ*. 2009; **339**: 279–82.

S37 Kitagawa K, Miwa K, Yagita Y, Okazaki S, Sakaguchi M, Mochizuki H. Association between carotid stenosis or lacunar infarction and incident dementia in patients with vascular risk factors. *Eur J Neurol*. 2015; **22**: 187–92.

S38 Sigurdsson S, Aspelund T, Kjartansson O, et al. Incidence of Brain Infarcts, Cognitive Change, and Risk of Dementia in the General Population: The AGES-Reykjavik Study (Age Gene/Environment Susceptibility-Reykjavik Study). *Stroke*. 2017; **48**: 2353–60.

S39 Inzitari D, Simoni M, Pracucci G, et al. Risk of rapid global functional decline in elderly patients with severe cerebral age-related white matter changes: the LADIS study. *Arch Intern Med*. 2007; **167**: 81–8.

S40 Lautenbach DM, Christensen KD, Sparks JA, Green RC. Communicating genetic risk information for common disorders in the era of genomic medicine. *Annu Rev Genomics Hum Genet*. 2013; **14**: 491–513.

S41 Frisoni GB, Altomare D, Thal DR, et al. The probabilistic model of Alzheimer disease: the amyloid hypothesis revised. *Nat Rev Neurosci*. 2022; **23**: 53–66.

S42 Cheng CPW, Lam LCW, Cheng ST. The Effects of Integrated Attention Training for Older Chinese Adults With Subjective Cognitive Complaints: A Randomized Controlled Study. *J Appl Gerontol*. 2018; **37**: 1195–214.

S43 Innes KE, Selfe TK, Brundage K, et al. Effects of Meditation and Music-Listening on Blood Biomarkers of Cellular Aging and Alzheimer’s Disease in Adults with Subjective Cognitive Decline: An Exploratory Randomized Clinical Trial. *J Alzheimers Dis*. 2018; **66**: 947–70.

S44 Kwok TCY, Bai X, Li JCY, Ho FKY, Lee TMC. Effectiveness of cognitive training in Chinese older people with subjective cognitive complaints: a randomized placebo-controlled trial. *Int J Geriatr Psychiatry*. 2013; **28**: 208–15.

S45 Oh SJ, Seo S, Lee JH, Song MJ, Shin MS. Effects of smartphone-based memory training for older adults with subjective memory complaints: a randomized controlled trial. *Aging Ment Health*. 2018; **22**: 526–34.

S46 Pereira-Morales AJ, Cruz-Salinas AF, Aponte J, Pereira-Manrique F. Efficacy of a computer-based cognitive training program in older people with subjective memory complaints: a randomized study. *Int J Neurosci*. 2018; **128**: 1–9.

S47 Small GW, Silverman DHS, Siddarth P, et al. Effects of a 14-day healthy longevity lifestyle program on cognition and brain function. *Am J Geriatr Psychiatry*. 2006; **14**: 538–45.

S48 Smart CM, Segalowitz SJ, Mulligan BP, Koudys J, Gawryluk JR. Mindfulness Training for Older Adults with Subjective Cognitive Decline: Results from a Pilot Randomized Controlled Trial. *J Alzheimers Dis*. 2016; **52**: 757–74.

S49 Barnes DE, Santos-Modesitt W, Poelke G, et al. The Mental Activity and eXercise (MAX) trial: a randomized controlled trial to enhance cognitive function in older adults. *JAMA Intern Med*. 2013; **173**: 797–804.

S50 Silva NCBS, Gill DP, Owen AM, et al. Cognitive changes following multiple-modality exercise and mind-motor training in older adults with subjective cognitive complaints: The M4 study. *PLoS One*. 2018; **13**. DOI:10.1371/JOURNAL.PONE.0196356.

S51 Fabre C, Massé-Biron J, Chamari K, Varray A, Mucci P, Préfaut C. Evaluation of quality of life in elderly healthy subjects after aerobic and/or mental training. *Arch Gerontol Geriatr*. 1999; **28**: 9–22.

S52 Lautenschlager NT, Cox KL, Flicker L, et al. Effect of physical activity on cognitive function in older adults at risk for Alzheimer disease: a randomized trial. *JAMA*. 2008; **300**: 1027–37.

S53 Andrewes DG, Kinsella G, Murphy M. Using a memory handbook to improve everyday memory in community-dwelling older adults with memory complaints. *Exp Aging Res*. 1996; **22**: 305–22.

S54 Cohen-Mansfield J, Cohen R, Buettner L, et al. Interventions for older persons reporting memory difficulties: a randomized controlled pilot study. *Int J Geriatr Psychiatry*. 2015; **30**: 478–86.

S55 van Hooren SAH, Valentijn SAM, Bosma H, et al. Effect of a structured course involving goal management training in older adults: A randomised controlled trial. *Patient Educ Couns*. 2007; **65**: 205–13.

S56 Youn JH, Lee JY, Kim S, Ryu SH. Multistrategic memory training with the metamemory concept in healthy older adults. *Psychiatry Investig*. 2011; **8**: 354–61.

S57 Fairchild JK, Scogin FR. Training to Enhance Adult Memory (TEAM): an investigation of the effectiveness of a memory training program with older adults. *Aging Ment Health*. 2010; **14**: 364–73.

S58 Frankenmolen NL, Overdorp EJ, Fasotti L, Claassen JAHR, Kessels RPC, Oosterman JM. Memory Strategy Training in Older Adults with Subjective Memory Complaints: A Randomized Controlled Trial. *J Int Neuropsychol Soc*. 2018; **24**: 1110–20.

S59 Hoogenhout EM, De Groot RHM, Van Der Elst W, Jolles J. Effects of a comprehensive educational group intervention in older women with cognitive complaints: a randomized controlled trial. *Aging Ment Health*. 2012; **16**: 135–44.

S60 McEwen SC, Siddarth P, Abedelsater B, et al. Simultaneous Aerobic Exercise and Memory Training Program in Older Adults with Subjective Memory Impairments. *J Alzheimers Dis*. 2018; **62**: 795–806.

S61 Pike KE, Ong B, Clare L, Kinsella GJ. Face-name memory training in subjective memory decline: how does office-based training translate to everyday situations? *Neuropsychol Dev Cogn B Aging Neuropsychol Cogn*. 2018; **25**: 724–52.

S62 Scogin F, Storandt M, Lott L. Memory-skills training, memory complaints, and depression in older adults. *J Gerontol*. 1985; **40**: 562–8.

S63 Valentijn SAM, Van Hooren SAH, Bosma H, et al. The effect of two types of memory training on subjective and objective memory performance in healthy individuals aged 55 years and older: a randomized controlled trial. *Patient Educ Couns*. 2005; **57**: 106–14.

S64 Manenti R, Sandrini M, Gobbi E, et al. Strengthening of Existing Episodic Memories Through Non-invasive Stimulation of Prefrontal Cortex in Older Adults with Subjective Memory Complaints. *Front Aging Neurosci*. 2017; **9**. DOI:10.3389/FNAGI.2017.00401.

S65 Solé-Padullés C, Bartrés-Faz D, Junqué C, et al. Repetitive transcranial magnetic stimulation effects on brain function and cognition among elders with memory dysfunction. A randomized sham-controlled study. *Cereb Cortex*. 2006; **16**: 1487–93.
